# Continuous Spike–Waves during Slow Sleep Today: An Update

**DOI:** 10.3390/children11020169

**Published:** 2024-01-28

**Authors:** Annio Posar, Paola Visconti

**Affiliations:** 1 IRCCS Istituto delle Scienze Neurologiche di Bologna, UOSI Disturbi dello Spettro Autistico, 40139 Bologna, Italy; paola.visconti@isnb.it; 2 Department of Biomedical and Neuromotor Sciences, Bologna University, 40139 Bologna, Italy

**Keywords:** epilepsy, epileptic encephalopathies, CSWS, ESES, Landau–Kleffner syndrome

## Abstract

In the context of childhood epilepsy, the concept of continuous spike–waves during slow sleep (CSWS) includes several childhood-onset heterogeneous conditions that share electroencephalograms (EEGs) characterized by a high frequency of paroxysmal abnormalities during sleep, which have negative effects on the cognitive development and behavior of the child. These negative effects may have the characteristics of a clear regression or of a slowdown in development. Seizures are very often present, but not constantly. The above makes it clear why CSWS have been included in epileptic encephalopathies, in which, by definition, frequent EEG paroxysmal abnormalities have an unfavorable impact on cognitive functions, including socio-communicative skills, causing autistic features, even regardless of the presence of clinically overt seizures. Although several decades have passed since the original descriptions of the electroclinical condition of CSWS, there are still many areas that are little-known and deserve to be further studied, including the EEG diagnostic criteria, the most effective electrophysiological parameter for monitoring the role of the thalamus in CSWS pathogenesis, its long-term evolution, the nosographic location of Landau–Kleffner syndrome, standardized neuropsychological and behavioral assessments, and pharmacological and non-pharmacological therapies.

## 1. Introduction

Within the concept of continuous spike–waves during slow sleep (CSWS), we can find several childhood-onset heterogeneous conditions that share electroencephalograms (EEGs) characterized by a high frequency of paroxysmal abnormalities during sleep, which have negative effects on the cognitive development and behavior of the child. These negative effects may have the characteristics of a clear regression or of a slowdown in development. Seizures are very often present, but not constantly. The above makes it clear why CSWS have been included in epileptic encephalopathies, in which, by definition, frequent EEG paroxysmal abnormalities have an unfavorable impact on cognitive functions, including socio-communicative skills, causing autistic features [1,2,3,4,5,6], even regardless of the presence of clinically overt seizures. This unfavorable impact has been confirmed in brain functional imaging studies [7]. In other words, in epileptic encephalopathies, epileptic activity itself (evidenced by EEG paroxysmal abnormalities) contributes to the development of serious cognitive and behavioral disorders, regardless of what might be expected due to the underlying pathology (if any) alone [8]. However, there have also been authors who aimed at diminishing the importance of CSWS in the development of cognitive deficits [9]. The first pioneering report that pointed out the connection between continuous EEG paroxysmal abnormalities (spike and wave complexes) and the presence of cognitive disorders should be attributed to Kennedy and Hill, who in 1942 described a child affected by “dementia dysrhythmica infantum” [10]. Between the 1970s and 1980s, Tassinari et al. described, in a very detailed and modern way, the electroclinical entity characterized by an EEG with diffuse spikes and waves lasting for at least 85% of slow sleep, clinically associated with cognitive decline, seizures, and motor impairment [11,12]. This is the typical form of the “electrical status epilepticus during slow-wave sleep” (ESES) that has been included among the epileptic encephalopathies according to the International Classification of the Epilepsies due to marked EEG abnormalities, leading to a cognitive impairment that may cause a severe degree of disability [13]. Over the years, it has also been found that when EEG paroxysmal abnormalities recur less frequently than typical ESES, cognitive disturbances can appear, but in a more heterogeneous manner and tending to be less severe than typical ESES. According to several authors, the current trend is to consider a spike and wave index (SWI) > 60% as suggestive of CSWS [14]. Even according to the current classification of the International League Against Epilepsy, the 85% threshold of SWI is no longer mandatory for the diagnosis of CSWS [5]. However, the defining features and diagnostic criteria of CSWS, and in particular of ESES, are still a matter of debate. Further, for a long time, the terms CSWS and ESES have been used interchangeably, but currently, ESES mainly refers to the EEG pattern, while CSWS refers to the syndromic picture associated with ESES; even on this point, however, there is no unanimity of opinion [15]. In this paper, for simplicity, we will use these two terms interchangeably. The main purpose of therapy for CSWS should be to prevent or reduce cognitive deficits [16]. Data regarding epidemiology are scarce, but CSWS cases are assumed to be 0.5% to 0.6% of children with epilepsy followed at tertiary referral epilepsy centers [17]. It is probable, however, that this recurrence is underestimated [18]. Etiopathogenesis, when known, is heterogeneous. To give an idea, Sonnek et al. [19], in a large retrospective monocentric study (95 children), also including “near CSWS” patients (SWI = 40–85%), found a structural/metabolic etiology in 43.2% of cases and genetic alterations in 17.9%, while the etiology was unknown in 38.9%. Regarding the pathogenetic mechanisms, it has been hypothesized that the negative effects of continuous EEG paroxysmal abnormalities on cognitive functions and behavior may be related to the spike-induced damage of the synaptic homeostasis processes that occur physiologically in sleep and that are crucial during development. During sleep, homeostasis is favored by synaptic weakening/elimination following the synaptic strength increase during wakefulness. Alterations in synaptic strength are shown in EEGs through alterations of sleep slow-wave activity (SWA). During CSWS, there are no sleep SWA changes, which occur again after CSWS remission: this finding suggests a spike-related impairment of the synaptic homeostasis during sleep. All of this may impair cortical wiring, causing the neuropsychological dysfunctions that are typical of CSWS [20,21].

The CSWS phenomenon is generally considered as a childhood-onset condition affecting the age of development and which tends to disappear during adolescence [22], but exceptional cases of CSWS persisting in the adult age have been reported [23]. However, the cognitive long-term outcomes are often poor, particularly in cases with a longer CSWS duration [22].

Landau–Kleffner syndrome (LKS), otherwise known as acquired epileptic aphasia, deserves a separate discussion, although there is a current of thought that considers LKS as an electro-clinical framework within ESES [24]. In this nosographic entity, the core symptom is an acquired aphasia associated with continuous EEG paroxysmal abnormalities during sleep, localized bilaterally in temporal regions, and not a global cognitive deterioration as in ESES [25]; seizures are not present in all cases. The possibility of a switch from LKS to ESES suggests a rather close link between these two nosographic entities which, in our opinion, should remain separate, as LKS presents very distinct and peculiar electrophysiological (localization of prevailing sleep EEG paroxysmal abnormalities), clinical (prominent impairment of verbal language skills), and neuropsychological (poor decoding of sounds) characteristics. 

In this review, we will describe the clinics and EEGs of typical and atypical ESES, the etiology, the pathogenesis, the pharmacological and non-pharmacological treatments, and the prognosis; we will undertake the same for LKS. Since there is a wide overlap in the etiopathogenetic factors hypothesized to underlie ESES and LKS, this last topic will be addressed in a single section.

## 2. Materials and Methods

**Figure 1:**
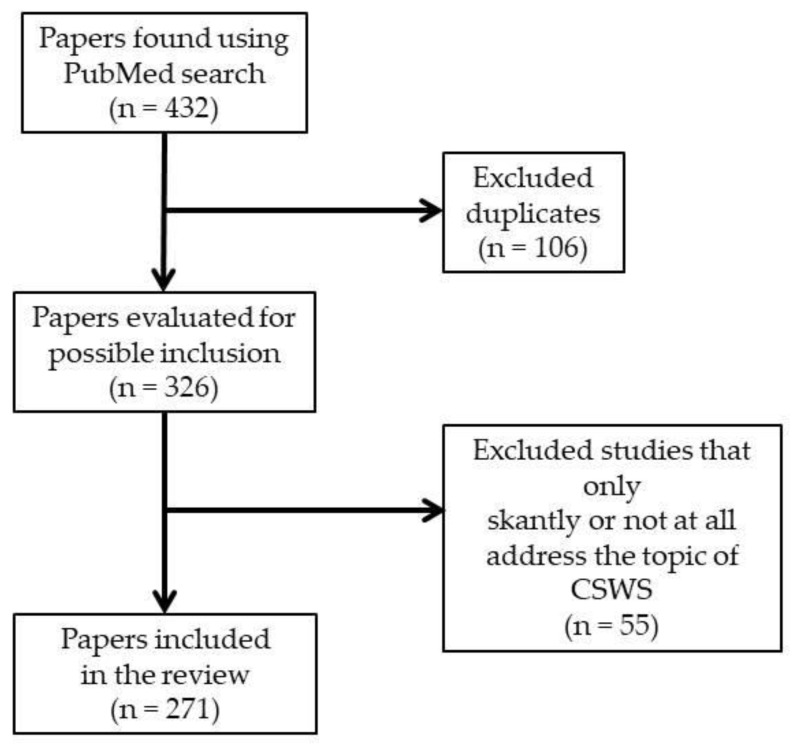
Paper selection flow chart.

We carried out a narrative review based on a paper search using the PubMed database (U.S. National Library of Medicine). We utilized two combinations of terms joined by the Boolean operator “AND”: “CSWS” AND “epilepsy”; “ESES” AND “epilepsy”; and also the term “Landau-Kleffner syndrome”. First, we considered all the articles found through these search terms that were published between June 2012 and May 2023. We found 131, 166, and 135 articles, respectively: after we removed the duplicates, the total number of articles was 326. We read all of these papers and excluded those that addressed scantly or not at all the CSWS topic or that were mere comments on other articles. Both authors of this paper contributed to the article selection. Finally, we included 273 papers in this review (Figure 1). We also considered some additional significant references, mentioned in the papers found using PubMed or known in some way by the authors. Both authors read all references included in this review.

## 3. Results

### 3.1. EEGs and Clinics of Typical and Atypical ESES

The core features of typical ESES, as described by Tassinari et al. [12], are (1) electrical-status epilepticus lasting for ≥85% of NREM (slow) sleep, documented by more than two EEG recordings during a period of ≥1 month; (2) different degrees of global cognitive deterioration, with decrease in full-scale IQ and performance IQ [26]; (3) behavioral disorders, including attention deficit, hyperactivity, aggressiveness, difficulty in social interaction, and (more rarely) psychosis (autistic behavior is also possible) [12,27,28,29]; (4) epileptic seizures, focal or apparently generalized, with a heterogeneous semeiology including absences; epileptic falls; clonic, tonic–clonic, partial motor, and complex partial seizures; and negative myoclonus (see atonic component for example at the upper limbs, showed through polygraphic recordings or restricted to lower limbs) can be associated in atypical absences [30,31,32]; it is possible that there are no overt clinical seizures [33], but this is an infrequent occurrence [19,34,35]; and (5) motor signs, including dyspraxia, ataxia, and dystonia. The described clinical EEG characteristics have substantially remained the same over the years [21]. Overnight EEG is considered as the gold standard for ESES diagnosis [36] because an ESES pattern may be recorded during the first sleep cycle of the night and may appear fragmented in subsequent ones. But in clinical practice, even an ambulatory EEG can be effective, according to several authors. For example, Nagyova et al. retrospectively studied 199 consecutive pediatric patients who underwent an ambulatory EEG. Suspected ESES was the motivation in 38.6% of cases, and this examination was useful in 97.5% of cases [37].

**Figure 2:**
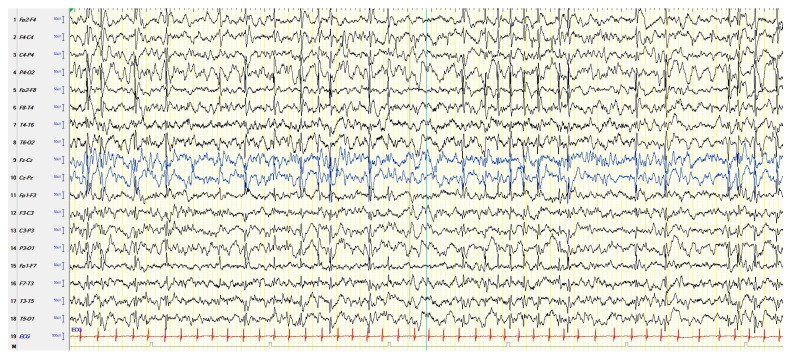
NREM sleep EEG in a boy aged 5 years and 7 months with autism spectrum disorder, intellectual disability, and focal epilepsy, showing subcontinuous diffuse spike–waves (lasting < 85% of NREM sleep), prevailing in the right posterior regions. NREM: nonrapid eye movement. EEG: electroencephalogram.

In the atypical forms of ESES, there is a great heterogeneity in the reported clinical features depending on the frequency and localization of the EEG paroxysmal abnormalities [38]. In fact, the localization of prevailing paroxysmal abnormalities is more heterogeneous than in the typical forms (focal, multifocal, unilateral, asymmetric, or symmetric bilateral), and diffuse paroxysmal abnormalities have been reported [39]. Figure 2 shows an example of atypical ESES.

Among the neuropsychiatric clinical features in atypical ESES, ADHD-like symptoms are prominent and seem to be related positively to SWI [40]. Acquired visual agnosia may be found in CSWS, as suggested in the case reported by Van Iterson et al.: a male aged almost 7 years presenting CSWS, prevailing in the left posterior regions, who lost the skill of recognizing familiar faces and naming pictures [41]. Kuki et al. reported acquired Kanji (morphogram) dysgraphia associated with visual processing impairment in two children with CSWS prevailing in the occipito-temporal region, in which functional neuroimaging showed a dysfunction located in the left posterior temporal lobe [42]. According to Tassinari et al. (also in individual cases), an adequate neurophysiological study aiming at localizing the “functional lesion”, associated with a detailed neuropsychological assessment, could help to analyze the cortical networks responsible for specific cognitive functions [43]. However, not all authors agree on the existence of a clear correlation between EEG picture and neuropsychological impairment, such as Pavlidis et al., who studied 24 children with idiopathic encephalopathy related to ESES (three had LKS). They found a lack of correlation between the severity/type of cognitive impairment and SWI and/or a topography of EEG paroxysmal abnormalities, suggesting the possible role of further factors during sleep (including an altered sleep homeostasis) in the neurobehavioral disorders [44]. It should not be forgotten that any structural brain abnormalities, if they are not the primary cause of the CSWS picture, can still influence its characteristics. For example, Mohammadi et al. described one child with hemi-ESES (that is, limited to one cerebral hemisphere) related to a congenital corpus callosum absence, inhibiting CSWS propagation from the right hemisphere to the left one. Their findings point out the focal origin of ESES as well as the corpus callosum’s role in the bilateral synchronous expression of EEG paroxysmal abnormalities [45]. On the other hand, the 85% SWI limit that separates typical forms of ESES from atypical ones may be of relative importance [5]. Caraballo et al., in a multicenter, retrospective long-term follow-up study including 117 CSWS cases, found similar electroclinical features comparing patients with a >85% SWI and patients with a <85% SWI [46]. Gencpinar et al. retrospectively analyzed the EEG recordings of 44 ESES/CSWS cases with a follow-up of at least two years in order to study (a) the SWI during NREM sleep EEG (>85% in 33 cases (typical ESES) and =50–85% in 11 (atypical ESES)) and (b) the maximum amplitude area of CSWS: anterior in 33 cases and posterior in 11. The authors found that the SWI rate (typical versus atypical ESES) as well as the maximum amplitude area of CSWS (anterior versus posterior) were not significantly related to clinical and brain imaging features or to the response to treatment (see seizure control and SWI reduction) [47]. Particularly in atypical forms of ESES, the technique called magnetoencephalography, utilizing superconducting quantum interference devices, can be useful in precisely localizing the spike source [48]. The diagnosis of CSWS, on the electrophysiological level, is therefore based on qualitative and quantitative criteria whose detection in the past was entrusted solely to the human eye. Over the years, increasingly precise and reliable automatic systems have been developed for the detection and quantification of spike–waves. These systems are very useful in CSWS, where EEG paroxysmal abnormalities are particularly frequent and their classic visual quantification can be very tiring [49]. Yu et al. proposed an SWI quantification neural network (SQNN) using a pre-labeling algorithm in order to quantify SWI automatically in children with ESES. The authors found that it would be possible to accurately and reliably quantify SWI through the SQNN, with a processing speed 100 times faster than that of experts [50]. However, to quantify the number of EEG paroxysmal abnormalities, SWI computing is not the only method: in fact, the number of spikes per unit of time can be also calculated. It should be borne in mind that the “time occupied by epileptiform activities” (i.e., SWI) and the “number of spikes per unit of time”, otherwise known as the “spike wave frequency” (SWF), differ substantially. Using the SWF, we can avoid the ceiling effect that is intrinsic to SWI calculation, especially when EEG paroxysmal abnormalities are more than 60 per minute, because SWI cannot exceed the limit of 100%, while SWF has no defined upper limit. Unfortunately, it is unclear whether the SWF or the SWI is more related to ESES clinical features [51,52]. In any case, the SWI is still the most used method today to quantify the number of EEG paroxysmal abnormalities. Not using the same method does not help with data sharing among CSWS researchers. Apart from this last important aspect, in general, the lack of studies sharing the same terminology concerning CSWS can be an obstacle to the reciprocal communication among clinicians and researchers dealing with this condition [53]. Still regarding the diagnosis on the electrophysiological level, Carvalho et al. showed that a wearable EEG device with only two bipolar channels for 24 h, through a semiautomatic template-match spike search, could be a good alternative, less expensive and better tolerated, to the full 10–20 long-term ambulatory EEG that is the classic tool for interictal spike quantification. In a clinical context, this alternative could be useful, particularly for the spike activity follow-up [54]. 

CSWS syndrome can begin as such, but very often it is the electroclinical evolution of another, more or less severe, epilepsy. For example, it can follow Ohtahara syndrome, a severe early-onset form of epileptic encephalopathy [55], but the most frequent occurrence is CSWS as the evolution of Rolandic epilepsy, otherwise known once as benign epilepsy with centro-temporal spikes [38] and today, with a more correct expression, as self-limited epilepsy with centro-temporal spikes (SeLECTS) (see also later). This is very important to keep in mind, as it shows us the possibility of changing from one apparently benign form of epilepsy to another with a less favorable prognosis. Further, CSWS may be the atypical electroclinical evolution of Panayiotopoulos syndrome, as suggested in two cases reported by Oguni et al. [56]. The recurrence of CSWS has also been described in a rare epileptic condition, sunflower epilepsy, a photosensitive and usually drug-resistant reflex epilepsy with seizures characterized by one’s head turning towards light, similarly to a sunflower, and a hand waving in front of the eyes [57]. The neuropsychiatric outcome is often disappointing [51,58,59]. Margari et al. carried out a long-term follow-up study in 25 CSWS children. At the onset of CSWS, 96% of the cases showed one or more neuropsychiatric disorders, including behavioral problems in 54%, intellectual disability in 37.5%, learning disorders in 33%, developmental coordination disorder in 17%, language disorder in 12.5%, and autism spectrum disorder in 8%. In the course of the follow-up, neuropsychiatric disorders persisted unaltered in 52% of the cases, worsened in 24%, and improved in 24%. Most of the cases without improvement in the follow-up had symptomatic CSWS [60]. 

How can CSWS interfere with cognitive functions? We know that spikes shown in EEGs correspond topographically to focal cortical areas of hypermetabolism according to the results of the positron emission tomography (PET) [61]; however, these metabolic alterations are not sufficient to answer our question. In healthy individuals, the physiologic overnight decrease in the slow-wave slope in NREM sleep is closely related to the sleep recovery function that is necessary for optimal cognitive performance. Starting from this assumption, Bölsterli Heinzle et al. retrospectively analyzed 14 CSWS patients, identifying the spike–wave “focus” as the area of the highest spike amplitude and the frequency of spike–waves as the SWI. They found no overnight change in the slow-wave slope in the “focus” are, while in “nonfocal” areas, the slow-wave slope decreased significantly. The spike–wave density (see SWI) was correlated with the overnight slope decrease impairment: the higher the SWI, the more impaired the slope decrease. The authors concluded by proposing that the overnight slope decrease impairment is a possible mechanism leading to CSWS neuropsychological deficits [62]. In another study, Bölsterli et al. carried out a retrospective analysis of overnight EEG in 10 children with idiopathic ESES. Throughout the night, the slope of the slow waves did not diminish significantly during ESES, especially at the focus of paroxysmal abnormalities, while after ESES remission, the slope diminished significantly. Compared to healthy controls, cases after ESES remission showed no significant difference in overnight slope decrease. The best cognitive outcome after ESES remission has been found in three cases who had some degree of slope decrease during ESES. These findings suggest that alterations in NREM-sleep slow waves induced by ESES are reversible, and the severity of cognitive impairment might be related to the severity of slow-wave impairment during ESES. Therefore, slow-wave analysis might be a prognostic factor for cognitive outcomes [63]. The longitudinal course of the slow-wave slope’s overnight change could be an objective EEG marker related to the cognitive function course [64]. Through a retrospective cross-sectional study considering 22 CSWS cases, Bölsterli Heinzle et al. found that the maximal spike–wave location, corresponding to the epileptic focus, was age-related and followed a posterior–anterior trajectory, similarly to various focal epilepsies. Therefore, younger cases should be more likely to show posterior foci than older ones. The authors hypothesized that this posterior–anterior trajectory of CSWS maximal spike–waves could be related to maturational modifications to the maximal expression of sleep slow waves [65]. Moreover, EEG paroxysmal abnormalities during NREM sleep, causing both an activation in epileptogenic zones (particularly in perisylvian and in prefrontal areas) and a deactivation of the default mode network (DMN) located beyond the epileptogenic area, may produce neuropsychological impairments [66]. For perisylvian network epilepsies, including idiopathic focal childhood epilepsies, ESES, and LKS, Halász and Szűcs proposed that a sleep-related alteration in physiological neural networks may underlie epileptogenesis. Homeostatic plasticity, which is a compensative process for cell loss recovery, may be associated with an excitability increase up to an epileptic level. In this way, physiological functioning derails to a pathological (epileptic) working mode. NREM sleep heightens epileptic processes, which in turn impair sleep functions. The vicious cycle between sleep and epilepsy works every night, altering brain functions, particularly during development. The type and degree of cognitive impairment should be related to the involved network’s function [67]. Ng and Hodges found that, in a comparison with patients with other types of epilepsy, children with recently diagnosed ESES turned out to be less impaired on the neurocognitive level than children with generalized epilepsy. This finding suggests that neuropsychological differences between ESES and other epileptic syndromes may develop as a long-term consequence of the neurological disorder and/or of pharmacological treatment [68]. However, within generalized epilepsies, there can be very heterogeneous situations in terms of severity and clinical characteristics, so much so as to make it difficult to interpret the data obtained by these authors [69]. Table 1 summarizes the clinical and EEG features of typical and atypical ESES.

### 3.2. Predictive Factors of the Evolution into CSWS

Considering the relevant impact of CSWS on cognitive neurodevelopment, it is very important to recognize, if possible, what the predictive factors of an evolution into CSWS are. Rolandic epilepsy is the prototype of benign epilepsies in childhood, but in some cases, it may have an atypical development, including CSWS. A retrospective study by Pesántez-Ríos et al. comprised nine patients with SeLECTS, all of whom showed atypical clinical features and CSWS. The average age at onset of Rolandic seizures was 5 years, while clinical and EEG deterioration occurred, on average, one and a half years later. Electroclinical features suggesting an atypical-development SeLECTS were the early onset of seizures; the appearance of new seizures with increased frequency; and the presence of an EEG fronto-centrotemporal focus, with increasing frequency, both in wakefulness and in sleep [70]. Porat Rein et al. carried out a retrospective cohort study involving 104 cases with SeLECTS. They identified the following risk factors for the development in these patients of severe atypical variants, including LKS and ESES: EEG spike–waves; EEG without evidence of left lateralization; and centro-temporal, frontal, or fronto-temporal EEG localization [71]. Subsequently, Porat Rein et al. developed a predictive model of the transformation of SeLECTS into epileptic encephalopathy with CSWS or LKS, collecting data from a cohort of 91 SeLECTS cases, of which 18 showed an encephalopathic transformation. The most important risk factors were the localization of EEG paroxysmal abnormalities in fronto-temporal and temporo-parietal areas and seizure semiology, including dysarthria or somatosensory auras [72]. But other studies were not limited to investigating the possible evolution into CSWS of SeLECTS alone. Desprairies et al. carried out a retrospective case–control study to search for any clinical or EEG feature indicative of later CSWS development at the time of the first seizure. They included 10 CSWS cases with available EEG at the time of the first seizure compared with 10 matched controls. They found no clinical or EEG features suggesting later CSWS development. However, during the follow-up, the occurrence of multiple types of seizures and seizure worsening were significantly more frequent in the CSWS cases [73]. According to Caraballo et al., clinical parameters predicting an evolution into CSWS were early-onset seizures, the appearance of new seizures, and a relevant increase in seizure frequency, while EEG parameters predicting an evolution into CSWS may be an increased frequency of the interictal EEG paroxysmal abnormalities during wakefulness and sleep, and the presence of bilateral spikes and waves [74]. Aeby et al., through a retrospective study, compared the awake EEG of 15 CSWS patients (including one with LKS: see later) with 15 matched cases with self-limited focal epilepsy (SFE). They found that, at the time of cognitive regression, CSWS patients were more likely than SFE cases to have a slow-wave index (SLWI) > 6%, an SWI > 10%, a cluster of spike–waves (CLSW) of ≥1 s, an SWF of >11%, and an EEG score of ≥3 (qualitative assessment: from grade 0 (normal) to grade 4 (maximally pathological)) [75]. Table 2 summarizes the clinical and EEG predictive factors of the evolution into CSWS.

### 3.3. Electrophysiology and Functional Neuroimaging

The Classic quantification of EEG paroxysmal abnormalities through SWIs is still useful and widely used today, but it has long had alternatives that have advantages over it (see Section 3.1). Furthermore, spike index semiautomatic quantification in CSWS is available today as a reliable alternative to the classic quantification based on visual scoring [76]. Peltola et al., by searching and averaging spikes in pre- and postoperative EEG recordings of 10 CSWS cases who underwent surgical treatment, found that calculating an integrated root mean square using multiple scalp electrodes during steady NREM sleep might provide a reliable parameter for spike strength evaluation in CSWS [77]. Ouyang et al., assuming that to explain the transition of brain activity to ESES is a challenge, used an S-estimator method (see the evaluation of regression coefficient) for analyzing multichannel EEG data in 11 ESES cases. The results confirmed the presence of a significant spike and global synchronization increase from wake to sleep. Further, global synchronization was strongly correlated with spikes [78]. Balaram et al. studied the overnight EEGs of 30 ESES children (SWI: ≥50%) through the analysis of spikes utilizing spatio-temporal mapping and electrical source analysis, coming to question misconceptions in the literature. They subdivided “generalised” (80%) ESES and focal (20%) ESES patterns. According to Balaram et al., the bisynchronous ESES subtype included in the “generalised” pattern (found in 10 cases) was due to the apparently synchronous bilateral spike activation with a tangential/oblique dipole (spikes were localized around the peri-Rolandic cortex). They considered the classic description of “diffuse” ESES spikes prevailing in the bilateral frontal areas as a misinterpretation related to the use of the 10–20 EEG system. Utilizing voltage mapping and source analysis, they identified an activation in the Rolandic cortex. The authors classified focal ESES as parietal, occipital, and temporo-occipital patterns, while (note!) they did not find a frontal ESES pattern [79]. De Tiège et al. studied the neurophysiological correlate of regional cerebral glucose metabolism in six children with CSWS (three with LKS and three with atypical Rolandic epilepsy) through a technique associating time-sensitive magnetic source imaging and positron emission tomography (PET) using fluorodeoxyglucose. In all cases, spike–wave onsets were associated with focal hypermetabolism, while the PA propagation to other regions was associated with focal hypermetabolism (five cases), hypometabolism (one case), or the absence of significant metabolic changes (one case). Probably, most of the hypometabolic areas were related to a remote inhibition mechanism [80]. Japaridze et al. studied, in 15 CSWS patients, the neuronal networks underlying the background EEG oscillations of this epileptic encephalopathy using two types of EEG analyses: the dynamic imaging of coherent sources and renormalized partial directed coherence. Regardless of epilepsy etiology, the background EEG pattern in CSWS patients was associated with a very complex network of coherent sources involving many cortical areas, the thalamus, and the cerebellum. Within this great network, the medial parietal cortex, precuneus, and thalamus play the role of central hubs that drive the information flow to other areas, in particular to the temporal and frontal cortex. This hierarchical network organization has ceased after successful treatment and therefore seems to be CSWS-specific [81]. Magara et al., using magnetoencephalography (MEG) in 16 non-lesional CSWS children, found dipole clusters located on heterogeneous cortical areas: the right Rolandic area (four cases), the right supramarginal gyrus (three cases), the left Rolandic area (two cases), the left supramarginal gyrus (one cases), the bilateral Rolandic area (three cases), and multiple anatomical areas (two cases). An earlier age at onset of epilepsy had the strongest negative prognostic effect on the intellectual level. In 12 cases, the long-term prognosis of the intellectual level was related to the fact that it was assessed at the time of CSWS diagnosis [82]. Li et al., using magnetoencephalography, assessed magnetic source activity in 55 children: 14 with SeLECTS and SWI ≥ 50%; 21 with SeLECTS and SWI < 50%; and 20 healthy controls. More severe cognitive impairment was present in cases with SeLECTS and SWI ≥ 50% compared to those with SeLECTS and SWI < 50% and to controls. Comparing the three groups, the authors found results suggesting that the deactivation of magnetic source activity located in the posterior cingulate cortex and in the medial frontal cortex could lead to cognitive impairment in SeLECTS [83]. Moeller et al. studied neuronal networks in 10 children with atypical benign partial epilepsy and SWI = 10–70% who underwent simultaneous EEG–functional magnetic resonance imaging (fMRI) recording. Several types of EEG paroxysmal abnormalities were recorded in all 10 children. The authors studied the individual paroxysmal abnormality-associated blood-oxygen-level-dependent (BOLD) signal changes in a single subject analysis for each paroxysmal abnormality type. They found focal BOLD signal changes that were concordant with the spike field (patterns similar to SeLECTS) as well as distant cortical and subcortical BOLD signal changes (similar to the patterns detected in CSWS). This finding suggests that childhood idiopathic focal epilepsies constitute a spectrum of overlapping conditions. Further, the group analysis showed a thalamic activation (see later regarding the hypothesis of a pathogenetic role of the thalamus) [84]. According to the review of Siniatchkin and Van Bogaert, in recent decades, fMRI has contributed much to the understanding of the pathogenetic mechanisms underlying CSWS. MEG and EEG source reconstruction showed sources of pathological brain activity associated with paroxysmal abnormalities in the perisylvian region. PET showed hypermetabolism in perisylvian, superior temporal, inferior parietal, and central cortex areas, which were related to paroxysmal abnormalities. The diffuse hypometabolism found in regions belonging to the DMN (see prefrontal and posterior cingulate cortices, parahippocampal gyrus and precuneus) could be due to a remote inhibition following epileptic activity. The aforementioned metabolic changes disappeared after CSWS recovery. The EEG-fMRI technique showed characteristic findings of epileptic encephalopathy: positive changes in blood-oxygen-level-dependent (BOLD) signals were found in the perisylvian regions, prefrontal cortex, anterior cingulate, and thalamus, while negative changes in BOLD signals were found in the DMN regions. The activation pattern represents a diffusion of epileptic activity, which is not dependent on etiology and the associated seizure type [85]. 

CSWS-associated high-frequency oscillations (HFOs) are hypothesized to be related to alterations in higher brain functions. Toda et al. studied the mechanisms generating HFOs in CSWS by analyzing the effects of diazepam (DZP) intravenous (IV) injections in three cases with CSWS previously treated with IV DZP. They performed time–frequency power spectral analysis on the spikes and compared the peak power and frequency of HFOs before and after IV DZP. The recovery of the HFOs’ peak power levels lagged behind the recovery of the spike amplitudes. Also, within 25 min after IV DZP, the HFOs’ power levels were lower than the baseline levels. The authors found no consistent modifications in the HFOs’ spectral frequencies. They hypothesized that dissociation in terms of recovery after IV DZP between spike amplitudes and the HFOs’ power might be due to different pathophysiological mechanisms generating spikes and HFOs [86]. Ohuchi et al., through a semi-automatic tool, examined ripple-band (80–200 Hz) HFOs in the EEG of 94 children with various epilepsies characterized by spikes activated in sleep, including CSWS. They found in the initial EEG that the median ratio of ripples per spike was significantly higher in the idiopathic CSWS cases than in those with SeLECTS, Panayiotopoulos syndrome, other focal epilepsies, and focal spikes without clinical seizures. The authors concluded by hypothesizing a close relation between the ripples’ dense generation and CSWS pathophysiology [87]. HFOs could be an electrophysiological parameter that is useful from a clinical perspective: according to Cao et al., the persistence of interictal scalp EEG HFOs after steroid therapy in CSWS children can predict seizure relapse and an unfavorable cognitive outcome [88]. According to Gong et al., HFO prevalence might somehow reflect epileptic activity. In fact, they found that 12 CSWS cases with HFOs tended to show more often epileptic negative myoclonus, atonies, myoclonus, and atypical absences than 9 cases without HFOs [89]. Further, HFOs might be considered as markers of seizure-onset zones from a surgical perspective, and at the same time, they seem to be related to the functional disruption of brain networks in CSWS [90]. 

In general, while NREM sleep facilitates the spread and propagation of paroxysmal EEG abnormalities, REM sleep has a suppressive effect on them. The inhibitory effect on paroxysmal abnormalities is mainly due to the phasic REM (PREM) microstate with respect to the tonic REM (TREM) microstate. All of this also applies to ESES, as demonstrated in the study by Giacomini et al. involving eight patients with ESES. Finding out the reason for the protective effect of PREM sleep on paroxysmal abnormalities could be very important for the development of new therapeutic approaches [91].

Auditory discrimination is very important for language development and for school learning. In a retrospective study including 14 children with typical SeLECTS and nine children with atypical SeLECTS, Filippini et al. showed that CSWS impairs central auditory discrimination, assessed through mismatch negativity (central evoked potential considered as the most sensitive auditory sensory accuracy marker), and may produce long-lasting negative effects on academic skills [92]. 

Table 3 summarizes the electrophysiology and functional neuroimaging findings in CSWS.

### 3.4. Treatments Regardless of Neurosurgery

The treatment of CSWS should have the improvement of EEGs, particularly during sleep, as its main target, as this is what affects the neuropsychiatric outcome even more than seizures (which may not even be present) [93]. Unfortunately, little evidence guides ESES treatment as, concerning this topic, mostly retrospective studies, case reports, or numerically limited patient series can be found in the literature [94]. Treatment options for ESES are essentially represented by antiseizure medications (ASMs), steroids (corticosteroids and ACTH), immunoglobulins, a ketogenic diet, and (see the following paragraph) surgery [95]. In these conditions, conventional ASMs prove to be ineffective in most cases, but in particular, corticosteroid treatment, also combined with ASMs [96], has caught on widely [18,58,97,98,99], leading to cognitive improvements [100]. There is no full agreement on the best treatments to be administered [101]. The drug resistance of this condition can lead to the concurrent use of several ASMs, but this represents a further risk factor, as the use of polytherapy appears to be related to cognitive deterioration [102]. 

Van den Munckhof et al., based on their pooled analysis of data from the literature considering 575 cases, suggested a higher efficacy (i.e., improvement in cognition or EEG) of steroids (81%) and neurosurgery (90%) versus ASMs (49%) and benzodiazepines (BZDs) (68%) [103]. Zhang performed a meta-analysis on the effects of hormones associated with ASMs versus ASMs alone in children with encephalopathy related to ESES. The study included 403 cases treated with hormones plus ASMs and 402 cases treated with only ASMs. Zhang found that the association of hormones and ASMs shows better effects than only ASMs regarding seizures, EEG, and cognition, and is relatively safe; this meta-analysis, which took into consideration a large population of patients, suggests that treatment with common ASMs is often ineffective in CSWS [104]. In a systematic review about the effects of pharmacological interventions in children with CSWS syndrome, Moresco et al. found no complete studies to include. They concluded that there was no evidence to support or refute the pharmacological treatment use for CSWS syndrome, and therefore, well-designed, randomized, controlled trials are necessary [105]. Sonnek et al., in their large retrospective monocentric study (95 children), also including “near CSWS” patients (SWI = 40–85%), found that CSWS was drug-resistant in over 70% of cases and that the most effective treatments were steroids and neurosurgery [19]. 

According to Kanemura et al., in CSWS epilepsy, characterized by secondary bilateral synchrony (SBS), the suppression of this EEG phenomenon might be necessary to avoid progressive neuropsychological dysfunction. The authors prospectively studied levetiracetam’s (LEV) efficacy on SBS, seizure frequency, and neuropsychological deficits in 11 children affected by drug-resistant epilepsy with SBS including CSWS. Eight cases (72.7%) were responders for SBS and for clinical seizures. Seven of these eight cases showed reduced hyperactivity and impulsivity [106]. Chen et al. retrospectively analyzed LEV’s efficacy in the treatment of 71 ESES patients. In 35 out of 50 cases (70%) showing seizures, there was a >50% decrease in seizure frequency. In the first 3–4 months of LEV treatment, there was a favorable EEG response in 32 out of 71 (45%) cases, with EEG normalization in five. ESES relapse occurred in eight out of 32 (25%) initial EEG responders. At the last follow-up, 47 cases (66%) still showed ESES, and only 13 cases (18%) regained their baseline function level. The authors concluded that LEV’s efficacy on EEG and on neuropsychological levels was limited on the whole [107]. According to Su et al., in 15 children with SeLECTS and ESES, LEV combined with short-term clonazepam can be effective on both EEG and seizures, with few side effects [108]. But in this retrospective study, as in most of the others mentioned here, data relating to the long-term effects of drug therapy are lacking. And unfortunately, as suggested in the study of Wiwattanadittakul et al. involving 33 children, the likelihood of a relapse of ESES for both steroids and benzodiazepines is high [109]. The choice of how to treat epilepsy with CSWS should be considered on a case-by-case basis. Not only the high frequency of drug resistance in CSWS should be taken into account, but also the possibility of relapse after a remission phase. Yuan et al. retrospectively analyzed eight CSWS children (follow-up: 6 months to 4 years), five of which showed brain lesions. After treatment with valproate (eight cases), clonazepam (eight cases), lamotrigine (four cases), and hormones (eight cases: continuous-pulse IV methylprednisolone (MP) for 3 days, followed by oral prednisone) for 3 months, seven cases showed a significant clinical and EEG improvement. Unfortunately, at 6 months, two cases relapsed [110]. Also, for relatively new ASMs such as topiramate (TPM), relapse rates in CSWS are high: Vrielynck et al. retrospectively analyzed the results of TPM in 21 children with CSWS. Three months after TPM introduction, sleep EEG improved in 14 cases and normalized in four: 16 out of these 18 patients showed cognitive or behavioral improvement. One year after TPM introduction, 20 cases were still on TPM and 10 showed persistent EEG improvement, most of them with clinical benefits, while relapse occurred in nearly half of patients [111]. Kanemura et al. prospectively studied the effects of perampanel (PER) on EEG secondary bilateral synchrony (SBS) in adolescents with epilepsy that was resistant to ASMs, including levetiracetam. They considered as responders for seizures and EEG those who showed a ≥50% decrease, respectively, from the baseline seizure frequency and SBS in EEGs. All four CSWS cases included in the study were responders for seizures, while three of the four were responders for SBS in EEGs [112]. Yu et al. retrospectively studied the effects of a PER add-on treatment in 54 children with focal epilepsy and ESES. After 6 months of this treatment, ESES resolved in 29 cases (53.7%). A longer ESES duration increased the risk of PER failure [113]. Li et al., in a retrospective study on PER treatment in the epilepsy of children, found an efficacy rate of 72.73% on seizures in SeLECTS combined with ESES, but the effects on EEGs are not specified [114]. Grosso et al. prospectively studied the effects of lacosamide add-on therapy in eight children with drug-resistant CSWS. A 24 h EEG was performed every 6 months in all patients. A neuropsychological assessment was performed before lacosamide introduction and after at least 12 months of therapy. After 6 months of therapy, six out of eight cases were considered as responders, with one case as partial responder and one case as a non-responder. In three out of eight cases, their EEGs normalized. After at least 12 months of therapy, five out of eight cases were considered as responders, while the neuropsychological assessment showed a slight improvement in two out of eight cases [115]. Fatema et al. found, in a retrospective study, favorable effects of a midazolam drip in eight out of 10 patients with CSWS [116]. 

Among the various ASMs, one that, based on retrospective studies, has shown to be more effective against ESES (with favorable effects on seizures and with behavioral and cognitive improvement) is undoubtedly sulthiame (STM) as an add-on treatment [117,118,119]. However, the frequent and severe adverse reactions of this drug (including ataxia, headaches, anorexia, behavioral disorders, gastrointestinal disorders, and metabolic acidosis, among others) have greatly limited its use. Fine et al., in a retrospective study, found in six children (four with ESES and two with LKS) that acetazolamide (AZM), an ASM with pharmacologic properties similar to sulthiame, may be effective in CSWS. After AZM, three cases (two with ESES, one with LKS) out of six showed resolution of CSWS, while all cases showed decreased clinical seizures as well as a subjective improvement in communication abilities and in school performance. In five cases (three with ESES, two with LKS) out of six, a subjective improvement in hyperactivity and attention deficit was reported [120]. 

Chen et al. carried out a prospective study on the effects of continuous oral dexamethasone in 15 CSWS cases that were refractory (not responding to several ASMs and prednisolone). Based on clinical and EEG evaluations, seven out of 15 cases were considered responders. No serious or life-threatening side effects were reported [121]. In recent years, new methods of administering steroid therapy have been developed. Bast et al. analyzed, through a retrospective study, the effects of MP pulse therapy in a sample that also included CSWS or LKS cases. The treatment included four pulses with oral or IV MP administered every week for three consecutive days. After this phase, the intervals between the pulses increased. After four pulses, according to clinical and EEG criteria, 11 of 15 (73%) patients with CSWS or LKS were responders. Adverse effects were mild and transient [122]. Hempel et al. retrospectively studied the pulse–dose effects of prednisone on language and behavior in 17 ESES children. Improvement was observed in most cases (10 out of 17: around 59%) in either language or behavior, with a higher likelihood of low-IQ cases to show improvements [123]. Jauhari et al. performed a retrospective study on children with neurobehavioral deterioration showing EEG paroxysmal abnormalities during sleep. Cases were classified as (1) ESES when SWI during sleep was ≥50% or (2) sleep-induced epileptiform activity (SIEA) when SWI during sleep was ≥25% and <50%. Outcomes were assessed at a follow-up of 3 months. Eighteen children were included: seven with SIEA and 11 with ESES. All patients were administered IV MP pulses and, afterwards, oral steroids for 8 weeks. In SIEA cases, the median SWI was 40%, with paroxysmal abnormalities prevailing in anterior regions, while in ESES cases, the median SWI was 80%. The SIEA and ESES patients presented a similar neurobehavioral profile. The post-steroid behavior scores improved in 6/8 cases with ESES and in 5/7 with SIEA. The median SWI improved in both groups to <5% in SIEA and to 45% in ESES. The IQ/social quotient mildly improved in both the SIEA and ESES groups. The authors concluded that an SWI > 50% during sleep should not be considered as a limiting factor for steroid therapy [124]. Meng and Dai retrospectively analyzed the effects of three courses of pulsed MP in 56 children with drug-resistant ESES. Each course lasted 3 days and was followed by oral prednisone for 3 days. The pulsed MP showed a global response rate of 73% on seizures and 70% on the EEG SWI. Significant improvements were found in verbal performance and full IQ as well as in learning and behavior. Unfortunately, after 1 year, the overall recurrence rate was high, i.e., 29% [125]. Chen et al. retrospectively evaluated the effects of MP in 82 children with ESES: 49 with SeLECTS variants, 27 with epilepsy with CSWS, and six with LKS. All patients received three courses, each including IV MP for 3 days, followed by oral prednisone for 4 days. After three courses, prednisone was taken for 6 months. The total EEG effectiveness rate was 83%, and was 82% for SeLECTS variants, 81% for CSWS, and 100% for LKS. Seizures improved rapidly in all three groups. After a 1-year follow-up, the recurrence rates were quite high: 47% for SeLECTS variants, 59% for CSWS, and 50% for LKS [126]. Studies comparing the effect of various drug classes on CSWS offer interesting insights. Through a retrospective USA multicenter study, Baumer et al. evaluated, in 81 children, the clinical and EEG effects of current CSWS treatments, classified as follows: BZDs, steroids, other ASMs, and other therapies. The most frequently used treatments as first-line therapy were BZDs and ASMs (respectively, 62% and 27%). Patients showed significantly greater odds of clinical improvement using BZDs or steroids than using ASMs and significantly greater odds of EEG improvement with steroids than with ASMs. The authors concluded suggesting an earlier use of BZDs and steroids in these patients [127]. Kılıç et al. retrospectively studied 33 ESES children with a follow-up of at least one year. At first access, 90% of cases had seizures, while 10% showed only school failure. Heterogeneous etiologies were detected: asphyxia (six cases), hydrocephalus (two), polymicrogyria (one), and mesial temporal sclerosis (one). All children received at least two ASMs except one, who received only one. BZDs proved to be the most effective medications. At the end of the follow-up, 72.7% of patients were seizure-free, while 57.5% of patients showed complete SWI recovery during NREM sleep. However, the authors did not consider patients receiving steroids [128]. 

The choice of when to start treating CSWS is not always easy. A suggestion regarding this comes from Uliel-Sibony and Kramer, who carried out a retrospective study including 17 children with SeLECTS, an SWI > 30% (mean: 60%), and ADHD/attention deficit disorder. They found data suggesting that the most important parameter leading to the use of steroids or high-dose diazepam is a neuropsychological evaluation showing cognitive deterioration [129]. This underlines, once again, the importance of carrying out serial neuropsychological and neurobehavioral assessments in patients with CSWS. 

The good effects of steroids suggest a pathogenetic role of inflammation, but prolonged treatments with corticosteroids may lead to undesirable side effects, so alternative therapies targeting inflammation are necessary. Jyonouchi and Geng described a patient with ESES who was successfully treated using a combination of immunomodulating agents regardless of oral corticosteroids that have been discontinued due to side effects. The patient showed a 30%, 50%, and 100% reduction in the ESES with the sequential addition of three immune-modulating agents selected on the basis of monocyte cytokine profiles: respectively, anakinra, IV immunoglobulin, and sirolimus. Also, the patient’s speech and behavior gradually improved [130]. The ketogenic diet (KD), already used for a long time with often satisfactory results in epileptic encephalopathies, may theoretically be considered as a treatment for ESES due to its influence on the GABA systems and its inflammation-reducing action. The results of KDs in ESES are heterogeneous: 38 children were reported in six papers. Overall, 53% cases showed EEG improvement, but EEGs normalized in only 9%; 41% cases had a >50% seizure decrease and 45% showed cognitive improvement [131,132]. Ville et al. performed a monocenter retrospective study on 42 patients with steroid-resistant or -dependent epileptic encephalopathies, including 13 with CSWS, in whom they associated oral steroids with the KD. For at least 6 months, 8/13 cases with CSWS responded to the KD addition, which allowed for the discontinuation of steroids in the responders. Cases with steroid-dependent CSWS seemed to be the best candidates for KD used in combination with steroids [133]. KD can also be effective in cases with structural etiology [134]. 

The lack of effective pharmacological therapies that are free of severe side effects has led to research into the effects of innovative drugs on CSWS. Wilson et al. retrospectively studied the effects of amantadine in 20 patients with refractory ESES. The use of this drug was suggested by some reports of amantadine efficacy in refractory absences. The median treatment duration was 11.5 months; it was generally well-tolerated. The median post-amantadine SWI during slow-wave sleep (53%) was significantly lower than the baseline SWI (76%). Six (30%) cases showed ESES resolution. Most cases showed subjective cognitive, linguistic, or behavioral improvement [135]. 

At the moment, there are too little data in favor of the effectiveness in ESES of vagus nerve stimulation [136] and of transcranial direct current stimulation [137].

What is the best approach when treating CSWS? According to Kotagal, CSWS requires a prompt diagnosis and aggressive therapy. A close follow-up, including overnight EEG, is useful to evaluate treatment effects. Cases who do not respond to BZDs at high doses and/or to valproate should receive prednisone for 3 months. For cases who do not respond to steroids or who show steroid dependence, IV immunoglobulin could be another option. Drug-resistant cases should be assessed for a possible surgery treatment (focal resection) [138]. 

Finally, sometimes, unpredictable worsening of the CSWS clinical picture can occur, at least apparently related to pharmacological treatment, as demonstrated by the case of CSWS described by Samanta et al., who presented an absence status following oral intake of clobazam [139].

Table 4 summarizes the non-surgical treatments for CSWS.

### 3.5. Neurosurgical Treatment

In the past, the neurosurgical treatment of CSWS has had the objective of removing any focal cortical lesions considered to be the etiopathogenetic basis of ESES. But nowadays, it is known that this treatment can be considered even in the absence of brain lesions, in particular when it is possible to identify the epileptogenic cortical area that is at the origin of ESES. Furthermore, it should be borne in mind that in CSWS, contrary to what was once thought, the presence of generalized seizures, without evident signs of localization, is not an absolute contraindication for hemispherectomy [140]. Also, the presence of a genetic etiology nowadays no longer represents an absolute contraindication to a neurosurgical treatment [141]. 

Gröppel et al. compared 11 children with ESES before surgery (hemispherectomy) with 21 age-matched controls with epilepsy but without ESES. They calculated language quotients before and after surgery. Before surgery, severe developmental delays prevailed significantly in the ESES group (n = 9) compared with the controls. In the first group, after surgery, ESES remitted promptly in 10 cases out of 11, and a significant improvement in language was found. While children with ESES had significantly worse preoperative language skills than those without ESES, at the last follow-up after surgery, no significant differences were found compared to controls [142]. Wang et al. carried out a retrospective study on 11 children with ESES who underwent resective (seven cases) or disconnective (four cases) neurosurgery. Etiologies, when known, included cortical malformations, encephalomalacia and gliosis, porencephaly, and Rasmussen’s encephalitis. Before surgery, all cases showed developmental delays, nine out of 11 cases had motor deficits, seven out of 11 showed language delays, and three out of 11 had visual field defects. After neurosurgery, nine out of 11 children showed a reduced seizure frequency, eight out of 11 showed neuropsychological improvement, and nine out of 11 had ESES resolution. Children with poor outcomes after surgery showed, in addition to ESES, more severe preoperative comorbidities [143]. Marashly et al. studied 14 carefully selected ESES cases who underwent resective neurosurgery for epilepsy and ESES; 12 with magnetic resonance imaging (MRI) suggestive of perinatal suffering and two with normal MRI. In ten out of 14 cases, the surgical procedure was a hemispherectomy, while in the other cases, it was temporo-parieto-occipital disconnection, frontal lobectomy, parieto-occipital resection, and limited corticectomy. The authors found that resective neurosurgery was effective in most patients, leading to long-term seizure freedom, ESES resolution, and cognitive/behavioral functioning stabilization [144]. Alawadhi et al. reported five patients who showed CSWS resolution after the surgical resection of an inciting focal lesion. Remarkably, in three cases out of five, MRI showed no visible epileptogenic structural abnormalities of the brain. They concluded that in drug-resistant CSWS cases, an epilepsy presurgical workup should be considered [145]. Jeong et al. retrospectively analyzed the outcomes of nine children with drug-resistant ESES and a unilateral structural lesion who were treated with a functional hemispherectomy. After neurosurgery, ESES was eliminated in all six cases with available postoperative EEG during sleep. Developmental regression stopped after the hemispherectomy, even if no patient returned to the pre-ESES baseline [146]. 

Yokosako et al. retrospectively studied three cases with drug-resistant ESES without structural abnormalities who underwent a corpus callosotomy. After surgery, one patient showed complete ESES resolution and IQ improvement, while in the other two cases, EEG paroxysmal abnormalities were lateralized to one hemisphere and the SWI decreased with moderate development and seizure improvement. In these last two cases, from 6 months after surgery, the SWI increased again, but without developmental regression [147]. 

In conclusion, neurosurgical treatment for CSWS currently represents an option only in selected cases, but very consistent data suggest that, when feasible, it usually proves to be very effective in most cases, both at the EEG and neurodevelopmental level. Therefore, a presurgical workup should be considered in all cases with drug-resistant CSWS [145].

### 3.6. Evolution and Prognostic Factors

In general, the evolution of CSWS is often unsatisfactory, with a complete recovery on the cognitive–behavioral level after the cessation of the CSWS that is observed only in a minority of cases [6,44]. Yilmaz et al. studied 14 ESES patients followed-up for at least 2 years. During ESES, 12 patients showed a cognitive impairment involving school performance. After ESES, seven cases had intellectual disability of a heterogeneous severity. At the end of the follow-up, globally, 12 cases were seizure free, of which seven were still taking antiseizure treatments, while two cases still showed seizures despite antiseizure therapy; ESES was stopped in all cases and EEGs were completely normal in seven patients [148]. 

As regards the prognostic factors, a long CSWS duration and the presence of a cerebral lesion seem to be the most important factors related to an unfavorable outcome for cognition. In cases with normal brain MRI, the clinical phenotype at onset seems to be very important for the cognitive outcome: when the presentation is that of an atypical SeLECTS, the patients will usually show no major cognitive deficits [6]. According to van den Munckhof et al., who included 575 cases in their pooled analysis of data from the literature, a normal development before ESES onset, as well as normal neuroimaging findings, may be predictors of improved outcomes concerning cognition and/or EEG [103]. Research regarding the long-term follow-up of this condition is not very rich. Pera et al. retrospectively studied the long-term cognitive outcome in 25 CSWS children (mean follow-up: 13.5 years). Seven cases (28%) with nonlesional epilepsy showed a positive outcome; three cases (12%) had persisting motor deficits without cognitive impairment; and seven patients (28%) with long-lasting CSWS (mean: 28.1 months) showed a negative cognitive outcome. The cognitive outcomes did not change in six cases with structural or metabolic pathologies preceding their CSWS onset; finally, two patients (8%) showed a negative outcome regardless of the CSWS duration or the presence of other neurologic disorders preceding the CSWS onset. The authors concluded that CSWS syndrome’s long-term outcome seems to be influenced by the treatment response, CSWS duration, and underlying etiology [149]. Hegyi et al. carried out a long-term follow-up (average time: 7.5 years) retrospective study including 33 ESES children (15 non-lesional versus 18 lesional), with bilateral discharges lasting for ≥ 75% of NREM sleep. The seizure type variability, seizure frequency, and status epilepticus frequency were higher in lesional cases. Cognitive function impairment was more severe in the lesional cases [150]. Maltoni et al., in a retrospective study including 61 CSWS patients with an SWF during sleep of ≥25/min, found that the SWF was inversely correlated with full and performance IQs during CSWS. Further, a longer-lasting SWF ≥ 25/min was related to worse verbal IQ and performance IQ results after CSWS disappearance. But also, other variables could play a role in the neuropsychological outcome, including an earlier age at the first recording of an SWF ≥ 25/min, perinatal distress, pathologic neurologic examinations, and drug resistance to ASMs [34]. De Giorgis et al., in a retrospective study including 16 children with idiopathic CSWS, found that intellectual impairment was significantly higher in children with early-onset CSWS (before 6 years) and lower in those with later-onset CSWS (from 8 years onwards) [151]. 

According to Caraballo et al., in non-lesional cases, cognitive recovery after the end of CSWS depends on the initial regression severity and duration. The CSWS duration appears as the most important prognostic factor of the cognitive outcome. However, etiology plays a main role in the outcome [74]. Escobar Fernández et al., in their retrospective study including 25 children, found that CSWS remission was longer when the CSWS onset was later. Cognitive and behavioral outcomes were poorer in “secondary” cases (that is, with abnormal neuroimaging or psychomotor delays) and in those with earlier-onset and longer-lasting CSWS [152]. Saraf et al. conducted a retrospective study including 52 patients with CSWS (idiopathic: 19; symptomatic: 33). They found that at the 1-year follow-up, immune-modulating treatments (steroids and IV immunoglobulin) were effective against seizures and language, irrespective of whether the cases were idiopathic or symptomatic. They found some favorable predictive factors for language at the 1-year follow-up based on EEGs: a normal background in the baseline EEG, the presence of generalized spikes, the absence of frontal-negative spikes, and a relatively low SWF during sleep [153]. Öztoprak et al. retrospectively studied 48 children with ESES/CSWS, 21 with typical ESES (SWI > 85–100% in NREM sleep) and 27 with atypical ESES (SWI ≥ 50% and < 85% in NREM sleep); the median follow-up duration after ESES diagnosis was 57 months. The neurocognitive outcomes were unfavorable in 50% of cases. The unfavorable neurocognitive outcomes seemed to be related to symptomatic/structural etiology, an SWI ≥ 85%, an earlier age at ESES diagnosis, a longer ESES duration, and a longer interval between epilepsy onset and ESES [154]. As suggested by Gardella et al., EEG features should be correlated with cognitive and behavioral assessments, possibly considering not only the SWI during NREM sleep, but also other EEG parameters that may affect the clinical picture, including topography, patterns of spread, fluctuations in the paroxysmal abnormalities, epileptiform activity during wakefulness, the possible presence of focal slowing, EEG background organization, and a sleep architecture derangement [155]. Let us never forget that not only the evolution of EEG and neuropsychological tests administered to the affected individual, but also behavioral observations and parental reports in ESES children can play an important role in determining the evolution of the clinical picture (see the initial cognitive deterioration and the subsequent improvement) [156]. Ucar et al. retrospectively evaluated 33 BCETS children who developed ESES clinical and EEG parameters related to their prognosis. They found that, particularly among cases aged ≤ 8 years, a shorter time to ESES resolution, the presence of ESES remission, and seizure control were associated with a good prognosis [157]. In conclusion, according to Arzimanoglou and Cross, the issue of cognitive and behavioral outcomes should take into account the great complexity of this condition, considering not only the EEG evolution but also the underlying etiology and age at diagnosis [59]. 

Table 5 summarizes the main prognostic factors of CSWS.

### 3.7. Landau–Kleffner Syndrome (LKS)

#### 3.7.1. Clinics, Epidemiology, and Differential Diagnosis

LKS is characterized by acquired mixed (comprehension and production) aphasia, which begins at the age of 3–5 years, with poor decoding of verbal and/or non-verbal sounds [158,159,160]. Even today, the diagnostic process of LKS may not be easy, and this can cause a delay in diagnosis [161]. Seizures are present in the majority of LKS cases (around two-thirds of patients) and are semiologically heterogeneous: focal motor, tonic–clonic seizures, and atypical absences (e.g., with chewing gestures or with lip smacking). Behavioral symptoms are frequently associated: hyperactivity; an attention deficit; impulsiveness (leading up to an ADHD picture); irritability; aggressive behavior; in some cases, autistic-like symptoms; anxiety; and depression [27,29,93,160,162,163,164,165,166,167,168]. In LKS, autism could be due to the epilepsy itself, but the presence of genetic factors common to LKS and autism should not be overlooked [28]. Learning disorders, frequently in mathematics, are the rule in patients with LKS [164]. Sleep disorders may be associated [160]. Also, difficulty in maintaining posture has been reported [169]. Development is not always completely regular before the onset of aphasia: according to a study of Caraballo et al., before aphasia onset, developmental language disorders were present in 19 out of 29 LKS cases, while behavior disorders were present in 14 out of 29 LKS cases [162]. The core neuropsychological dysfunction in LKS seems to be a deficit of phonological decoding, involving not only verbal language. In fact, according to Lévêque et al., music perception skills also seem to be impaired in the form of high pitch discrimination thresholds and inadequate short-term memory for melody and rhythm. These findings suggest that, in LKS, beyond verbal impairments, the brain networks implicated in sound processing and encoding are severely altered [170]. 

Although epidemiological data are lacking, the condition is undoubtedly rare. According to Kaga et al., in Japan, the incidence of LKS individuals aged 5–14 years was about 1 in 1,000,000, while the prevalence of LKS individuals aged 5–19 years and under medical care was about 1 in 300,000–410,000 [171]. Furthermore, it cannot be ruled out that the real incidence of LKS is underestimated due to the incomplete awareness of this condition. Yet, for a better outcome, the awareness of LKS is very important, as it favors an early diagnosis and the prompt start of treatment [172,173].

A multidisciplinary approach appears to be the most proper for the diagnosis and treatment [174]. Concerning differential diagnosis, it is crucial to rule out, in children with aphasia, a large group of heterogeneous pathological conditions, including, primarily, hearing loss, autism spectrum disorder (ASD), or ADHD [160,175]. In particular, for ASD, a differential diagnosis could be difficult due to the early language regression reported in a large number of ASD children and the frequent finding of EEG paroxysmal abnormalities in ASD. On the other hand, it should be borne in mind that aphasia can be due to various organic causes, including space-occupying brain lesions, post-traumatic sequelae, and infections [160,176]. A differential diagnosis has to be carried out with other epileptic syndromes characterized by a marked increase in EEG paroxysmal abnormalities during sleep, including ESES (typical and atypical) and Lennox–Gastaut syndrome [160]. Finally, metabolic disorders such as mucopolysaccharidosis type III, due to a lysosomal enzyme deficit in the heparan sulfate catabolism, which is characterized by behavioral disorders, decreased verbal communication, and only subtle somatic signs, should be also considered in differential diagnoses [177]. Evidently, for a correct differential diagnosis, EEG during sleep is of crucial importance.

#### 3.7.2. Neurophysiological Findings

EEG recordings show spikes and spike–waves prevailing in the posterior temporal regions, bilaterally, much more diffuse and frequent during non-REM sleep, when they become continuous or subcontinuous. Background activity during wakefulness and sleep is normal [158,159,160]. As suggested by the three cases reported by van Bogaert et al., EEG during sleep may be normal in the early language regression phase, so it should be repeated when the clinical suspicion of LKS is strong [178]. Discriminating frequency modulation changes within speech is crucial for phoneme detection, and therefore, for language comprehension. The frequency-modulated auditory-evoked response (FMAER) technique assesses the processing of rapid frequency modulation within an auditory stream performed by the superior temporal gyri and contiguous cortex. Patients with receptive language deficits, including individuals with LKS, show absent left or bilateral responses [179].

#### 3.7.3. Etiology

Etiology is not known in most cases. Brain lesions are rare and unspecific. Genetic factors have recently been called into question. In particular, mutations of the GRIN2A gene (16p13.2) have been found in some cases with LKS [180]. The GRIN2A gene encodes for the GluN2A protein, a subunit of the N-methyl-D-aspartate (NMDA) receptor. High concentrations of the GluN2A protein are present in cerebral regions that are critical for language. In recent years, the concept of common genetic factors for SeLECTS, CSWS, and LKS has caught on [181,182], further indicating a link between these nosographic entities. The topic of the etiopathogenetic factors of LKS will be further addressed later, together with ESES (see Section 3.8).

#### 3.7.4. Physiopathology

Clinical, neurophysiological, and brain glucose metabolism findings suggest that EEG paroxysmal abnormalities play a crucial role in cognitive impairment by meddling in neuronal networks, both at the epileptic focus site and at connected (and far) areas. Consequently, therapy should have the suppression of EEG paroxysmal abnormalities as the target [160,183].

Using 99mTc-ECD SPECT imaging, asymmetrical temporo-parietal perfusion seems to be a common finding in LKS [184]. Pullens et al., through the fMRI of a recovered adult LKS female, studied audiovisual multi-sensory processing, because LKS individuals are often skilled in reading, but fail in speech perception. They found data compatible with undamaged temporal lobe processing and with impaired connectivity between the temporal and frontal lobes, which could be one pathogenetic mechanism underlying LKS [185]. Also, the nondominant hemisphere may play an important role in language comprehension (see speech processing). Language acquisition would need a process of understanding the meaning of words by integrating visual, auditory, and contextual information. It has been hypothesized that the nondominant hemisphere works mainly through this integrating role [186]. As suggested by the case described by Datta et al., in LKS, there may be a cerebral reorganization of the language network to the right (nondominant) hemisphere, therefore leading to an atypical language representation. This phenomenon, however, is also present in other epileptic syndromes that involve the cortical areas responsible for language [187].

#### 3.7.5. Outcome

The outcomes of LKS are very heterogeneous. According to a retrospective study of Caraballo et al., at the end of a mean follow-up of 12 years, only eight out of 29 LKS cases recovered their language completely, while 21 cases still showed language deficits of different degrees [162]. Patients with LKS could need life-long support due to persisting and potentially debilitating deficits of verbal communication [188]. In a retrospective study involving 11 children, four cases showed no language problems after adolescence, four cases had moderate language problems, and three cases presented a severe language impairment > 10 years after the diagnosis [189]. A positive outcome was associated with late-onset aphasia, short-lived initial receptive manifestations, and speech performances characterized by marked fluctuations. Seizure prognosis is good, as they remit within adolescence [160].

Table 6 summarizes Section 3.7.

### 3.8. Treatments for LKS

In the above-mentioned systematic review of Moresco et al., also concerning pharmacological treatments for LKS, no completed studies to include were found, and therefore, well-designed, randomized, controlled trials are necessary [105]. In order to improve the prognosis, treatment should start as soon as possible. If seizures are present, several ASMs may be effective, and usually, seizure control is easy to achieve. Drugs such as valproate, clobazam, levetiracetam, and ethosuximide, considered as “spike-suppressing”, are preferred [160]. Also, AZM might be an effective treatment: see above the retrospective study of Fine et al. [120]. Instead, there are some drugs such as carbamazepine, oxcarbazepine, phenytoin, and phenobarbital that, in LKS, should not be used due to a possible exacerbation of EEG paroxysmal abnormalities [160]. The most effective treatments for the EEG picture, and consequently, for language and behavior, are corticosteroids (e.g., oral prednisone or oral prednisolone) [98,160], suggesting an underlying inflammatory pathogenetic mechanism. On the other hand, there are insufficient elements in favor of the use of IV immunoglobulin in these patients [160]. Immunotherapy (oral steroids and IV immunoglobulin) should be a therapeutic option, particularly in LKS patients with GRIN2A mutations [190]. Combined treatment with pulse BZD and corticosteroids could be very useful on both the clinical and EEG level in LKS according to Devinsky et al. [191]. There are limited data in favor of the favorable effects of a KD in individuals with LKS [132]. 

Multiple subpial transections (MSTs) (that is, the transection of only the horizontal corticocortical fibers and not of the vertical cortico-subcortical ones) in the posterior dominant temporal lobe is a surgical technique aimed at interrupting epileptic seizures without damaging the eloquent cortex. According to some authors, MST might lead to the recovery of language skills in LKS children [192]. However, this surgical technique should be restricted to drug-resistant patients and cases with steroid dependency or toxicity. But unfortunately, there are relatively few reliable data concerning the behavioral outcome of surgery for epilepsy (in general, not only for LKS) [193]. Downes et al. studied the effects of the long-term outcomes of MST on children with drug-resistant LKS or other ESES-related regressive epilepsies. Further, they explored predictors of such outcomes. They included 14 cases who underwent MST of the posterior temporal lobe compared with 21 cases who underwent only presurgical investigations and not surgery. At follow-up, no significant differences were found between the two groups with regard to language, nonverbal skills, adaptive behavior, or quality of life. The authors concluded that there is insufficient evidence for the significant benefits of MST in these conditions [194]. Fine and Nickels reported a girl with drug-resistant LKS, in which the seizure focus was localized in the right parietal cortex. The child underwent right temporo-parietal resection, after which she remained seizure free, her EEG normalized, and her language was recovered [195]. According to the recent systematic review of Hajtovic et al., there is limited evidence regarding the effects of vagus nerve stimulation (VNS) on seizure, cognitive, and behavioral outcomes in children with LKS [196]. 

Obviously, speech therapy is also indicated in children with LKS: it consists of several patient-centered interventions that usually include augmentative and alternative communication techniques aimed at improving speech. In younger patients, the treatment plan should also include psychomotor therapy [160]. In particular, for older children, cognitive linguistic treatment could be useful to improve spoken language. However, more research is necessary to optimize speech therapy in children with LKS [197]. The data in the literature on rehabilitation or speech therapy in LKS are even more sparse than those on pharmacological or surgical treatment [198].

Table 7 summarizes the treatments for LKS (pharmacological and non-pharmacological).

### 3.9. Etiopathogenetic Factors of CSWS (Including ESES and LKS)

CSWS is a very heterogeneous condition that may be related to genetic factors as well as to congenital or acquired cerebral lesions [145]. A large number of heterogeneous brain structural alterations have been reported in subjects with CSWS, including polymicrogyria (in particular, unilateral), migration disorders, perinatal hypoxic–ischemic encephalopathy, hydrocephalus, schizencephaly, porencephalic lesions, encephalitis, and intracranial hemorrhage [199,200]. Note that an EEG pattern closely resembling CSWS has also been reported in a mouse model of focal cortical dysplasia [201]. EEG patterns of ESES have been reported even in patients with congenital Zika virus syndrome [202]. He et al. studied resting-state functional fMRI in nine cases with SeLECTS and ESES, in 17 with SeLECTS but without ESES, and in 36 healthy subjects. They found that cases with SeLECTS and ESES, compared with others, showed decreased whole-brain functional connectivity in the salience network (otherwise known as the midcingulo-insular network) or in the central executive network (otherwise known as the lateral frontoparietal network), but not in the DMN (otherwise known as the medial frontoparietal network). These findings may lead to a better understand of the underlying pathogenetic mechanisms in cases of SeLECTS with ESES [203].

Growing data from the literature suggest an involvement of the thalamus in the pathogenesis of CSWS [17,204,205]. Neonatal thalamic lesions are not rarely described in CSWS. Losito et al., studying a sample of 60 CSWS children with early thalamic injury, found that cases evolving into typical ESES (that is, occupying > 85% of slow sleep) more frequently had damage in the mediodorsal thalamus and bilateral cortico-subcortical injury; moreover, cases with typical ESES showed the most severe behavior and cognitive impairment in the acute stage. Ischemic stroke, hemorrhagic infarction, and hydrocephalus shunting were associated with behavioral disorders [206]. Leal et al. reported nine cases with unilateral neonatal lesions of the thalamus that evolved into CSWS. A loss of thalamic volume prevailed in the medial and dorsal nuclei. Thalamic lesions were associated with white matter loss and ventricle enlargement in the same hemisphere. Cortical thickness quantification showed no hemispheric asymmetries. The impact on physiological EEG rhythms was mild. CSWS were lateralized to the lesioned hemisphere. Unilateral selective thalamic–cortical disconnection was therefore associated with a focal pattern of CSWS in patients with unilateral neonatal thalamic lesions [207]. Also, neonates with thalamic hemorrhage due to straight sinus thrombosis, without widespread cerebral damage, are predisposed to develop typical or atypical ESES disorder [208]. Öztürk et al. retrospectively studied subcortical gray matter volumes in a sample of 15 children with ESES, all with MRI considered normal. They found that the total relative volume of the thalamus was significantly lower in ESES cases than in 30 healthy controls and 15 cases with SeLECTS. Both the right and left relative volumes of the thalamus were lower in ESES cases than in healthy controls and cases with SeLECTS. This study suggests the possible involvement of the thalamus in ESES pathogenesis, even in the absence of detectable lesions in MRI [209]. A pathogenetic role of the thalamus can also be hypothesized in cases of CSWS with cortical malformation. Bartolini et al. carried out a prospective follow-up study involving 27 cases with polymicrogyria and CSWS. Using a comparative volumetric analysis, they found significantly smaller volumes of ipsilateral thalami and polymicrogyric hemispheres in patients with polymicrogyria and CSWS compared to cases with polymicrogyria without CSWS, cases with SeLECTS, and controls with headaches [210]. Van den Munckhof et al. studied neuroimaging findings in a sample of individuals with perinatal thalamic injury. They found in the prospective cohort (23 cases) that perinatal thalamic injury was followed by ESES in the majority (about 83%) of individuals after a mean follow-up of 8 years. The thalamic volume at 3 months was correlated to neurodevelopmental outcomes (see IQ/developmental quotient) [211]. Carvalho et al., in their retrospective study, found structural lesions in 25 out of 53 CSWS patients, which included an early thalamic injury in almost all cases (24/25). Most thalamic lesions were localized in one hemisphere, corresponding in all patients to CSWS lateralization [35]. Sánchez Fernández et al., in their case–control study, found a small relative thalamic volume in 18 ESES children with normal neuroimaging in comparison with 29 subjects with refractory epilepsy and 51 healthy individuals, controlling for age and total brain volume [212].

Functional thalamic abnormalities may also be present in cases with normal thalamic structures. Agarwal et al. retrospectively analyzed thalamic glucose metabolism in 23 CSWS children (with normal thalami based on MRI) through F-18-fluorodeoxyglucose (FDG)-positron emission tomography (PET), considering the standardized uptake value normalized to the whole brain (nSUV). Abnormalities in the thalamic glucose metabolism were found in 18 cases (78.3%). The thalamic nSUV was lower (six cases) or higher (one case) bilaterally in seven children without asymmetries. Abnormal thalamic symmetry was found in 11 cases, of which six showed a unilateral thalamic abnormality (three with higher metabolism and three with lower metabolism), while five children showed an abnormal asymmetry index with bilaterally normal (four cases) or higher (one case) thalamic metabolism [213]. Using magnetic resonance spectroscopy, Kilic et al., in a case–control study, found a significantly low NAA/Creatine ratio in 21 patients with ESES, which is indicative of neuronal cell loss/dysfunction, bilaterally in the thalamus, a structure that seems to be normal in MRI [214]. Leal, through a transversal exploratory study, using high-resolution EEG carried out in five CSWS children with perinatal thalamic hemorrhages localized in one hemisphere, found that perinatal thalamic lesions may produce, years later, a focal onset of paroxysmal abnormalities with characteristics of ESES in a cortex without demonstrable lesions, leading also to a prominent secondary propagation of paroxysmal abnormalities [215].

But there are also data that do not seem to be in line with an involvement of the thalamus in the pathogenesis of CSWS. In a retrospective study, Ligot et al. performed positron emission tomography through [18F]-fluorodeoxyglucose during wakefulness in 17 children affected by cryptogenic CSWS, finding hypermetabolism in bilateral perisylvian areas and hypometabolism in the following areas: the prefrontal cortex, precuneus, posterior cingulate cortex, and parahippocampus. The functional connectivity between hyper- and hypometabolic areas was altered. They found no thalamic metabolic alterations, suggesting a primary cortex role in CSWS genesis [216].

Even apart from lesions specifically of the thalamus, CSWS may occur after a perinatal stroke. In a cross-sectional study including 43 patients globally, Azeem et al. found that a higher EEG spike frequency and delta power during NREM sleep may be EEG biomarkers of the risk of developing ESES risk in subjects with perinatal stroke [217]. ESES can also be the long-term consequence of neonatal cerebral sinovenous thrombosis [218]. Symptomatic CSWS cases include also those with early-onset hydrocephalus; in these patients, EEG paroxysmal abnormalities would hypothetically be related to the shunt or to the possible presence of associated cortical or subcortical lesions [219]. The case reported by Taskin et al. suggests a possible correlation between a previous treated herpes simplex virus (HSV)-1 encephalitis and CSWS [220], while that described by Hu et al. suggests a possible involvement of onconeuronal antibodies in the pathogenesis of CSWS [221]. Dedeoglu et al., using brain MRI, found no significant difference regarding the corpus callosum thickness between children affected by SeLECTS with (N = 15) and without (N = 53) ESES [222].

Inflammation could play an important role in the pathogenesis of CSWS. Van den Munckhof et al., retrospectively studying 11 ESES cases, found that serum inflammatory mediators, and in particular, interleukin-6 (increased during ESES, decreased after immunomodulating treatment), could be related with disease activity. A decrease in interleukin-6 was associated with clear EEG and neuropsychological improvement [223]. 

Ayça et al. found that, in CSWS children, their 9:00 a.m. melatonin levels were significantly lower than in patients with epilepsy and healthy controls [224]. Instead, Tarcin et al. found no significant differences in the median basal melatonin levels between 91 children with epileptic seizures and 21 children with ESES; however, the median basal melatonin levels were significantly lower in both children with epileptic seizures and in those with ESES compared with the controls [225]. ESES has also been associated with fetal alcohol syndrome [226,227]. In children with benign focal epilepsy, ESES, associated with cognitive deterioration, can be induced by old ASMs, such as phenytoin, carbamazepine, and phenobarbital, but also by a relatively new antiepileptic drug such as oxcarbazepine. In these cases, the therapy change is followed by a prompt electroclinical improvement [228]. 

Over the last few years, an increasing number of genetic abnormalities, each different from the other, associated with CSWS have been gradually described in patients with CSWS. Among the reported genetic conditions with a high recurrence of CSWS is dup15q (copy number gains of 15q11–q13): Arkilo et al. retrospectively found that EEGs showed CSWS in 15 out of 42 children (35.7%) with neurodevelopmental disorders due to dup15q; 13 of these 15 children also showed clinical seizures [229]. This anomaly is identifiable using array CGH, as well as 22q11.2 microduplication syndrome, another genetic condition frequently associated with CSWS [230]. Many other copy number variants, detected through array CGH and affecting several other chromosomes, have been described in CSWS (3q25 deletion, 3q29 duplication, 8p23.3 deletion, 10q21.3 deletion, 11p13 duplication, 16p13 deletion, and Xp22.12 deletion) [22]: we do not deem it useful to dwell on them. But frequently, mutations of single genes are involved in the etiopathogenesis of CSWS. In a minority of patients with LKS and CSWS (no more than 20% of cases), de novo or inherited in an autosomal dominant manner, heterozygous pathogenic mutations in the GRIN2A gene have been found. This gene encodes GluN2A, the α2 subunit of the NMDA excitatory glutamate receptor, which plays a very important role in the control of synaptic plasticity [44,231,232,233,234,235,236,237,238,239,240,241,242,243,244,245,246]. Also, mutations in several other genes have been implicated in the etiopathogenesis of CSWS: for example, the connector enhancer of kinase suppressor of Ras 2 (CNKSR2), which is located on the short arm of the X chromosome (Xp22.12) [247,248]; cyclin-dependent kinase-like 5 (*CDKL5*) gene, whose mutations are usually involved in early-onset epileptic encephalopathy with a severe neurodevelopmental disorder [249]; the Cysteinyl-tRNA synthetase 2 (*CARS2*) gene [250], which has been reported to be associated with severe myoclonic epilepsy, neuroregression, and complex movement disorders; and the *KCNQ2* gene, involved in benign familial neonatal seizures [251]. According to Gong et al., pathogenic or likely pathogenic mutations of other genes such as *KCNA2*, *SLC9A6*, *HIVEP2*, and *RARS2* are also involved in some cases with developmental and/or epileptic encephalopathy with ESES [245,252]. The most common phenotype reported in *DYNC1H1*-related epilepsy is represented by West syndrome, but also, one case with CSWS has been described [253]. Also, cases with a genetic basis usually show neurocognitive and behavioral worsening associated with CSWS, which is often difficult to identify because they usually have a neurodevelopmental impairment that precedes the onset of CSWS. Knowing the genetic diagnosis can sometimes suggest the most effective treatment. An example of this is given by the case recently reported by Russo et al., a girl showing encephalopathy with ESES and cerebellar signs, who presented a de novo *KCNA1* variant in the Kv-specific Pro-Val-Pro motif. Remarkably, this case had a persisting noteworthy electroclinical response to ACTH treatment [254]. The pathogenetic relationships between involved gene mutations and ESES are not always clear. For example, the SCN2A gene encodes Nav1.2, a voltage-gated sodium channel expressed in the brain. SCN2A gene mutations are associated with several epileptic syndromes including ESES. Interestingly, ESES can be associated with both the gain and loss of functional variants of the SCN2A gene [255]. ESES can also develop in patients with Christianson syndrome, an X-linked condition due to *SLC9A6* gene mutations characterized by a complex clinical picture, including intellectual disability, a lack of speech, autistic behavior, ataxia, acquired microcephaly, hyperactivity, and drug-resistant epilepsy [256,257,258,259]. Bhat et al. reported CSWS in one patient with Rett syndrome (with MECP2 gene mutation) and in another with both neurofibromatosis 1 and Lhermitte–Duclos syndrome [260]. We would mention another possible genetic etiology of CSWS: a mutation of the *WAC* (WW domain-containing adaptor with coiled-coil) gene (located on 10p12.1). *WAC* gene mutations are considered to be related to DeSanto–Shinawi syndrome, characterized by intellectual disability, behavioral disorders, and speech impairment [261]. An increased risk of epilepsy, including ESES, has been reported in Prader–Willi syndrome (PWS), a genomic imprinting disorder due to an absent expression of paternal genes located on chromosome 15q11.2-q13 [262]. Koolen–de Vries syndrome (KdVS), due to 17q21.31 microdeletions or pathogenic *KANSL1* gene mutations, is clinically characterized by congenital malformations, neonatal hypotonia, developmental delay, and facial dysmorphism. About half of cases show epilepsy. Khan et al. reported six KdVS children with CSWS: four of them received a diagnosis of epileptic encephalopathy with CSWS, while two of them were diagnosed with LKS. Two cases underwent a KD variation, and for both, a clinical improvement was reported. The authors concluded that a prolonged EEG during sleep should be performed in all KdVS children showing a regression or plateau of development, especially when there is a previous history of seizures [263]. Mowat–Wilson syndrome is a rare genetic disease due to a heterozygous deletion or function loss of the *ZEB2* gene located on chromosome 2. In a retrospective study, Bonanni et al. found anterior ESES with SWIs > 85% in all five cases with overnight sleep EEGs, and in three out of five cases, there was a related cognitive and motor regression. In two cases, a marked cognitive and motor improvement was observed when EEG paroxysmal abnormalities during sleep decreased. The clinical significance of ESES is difficult to assess in these cases due to severe intellectual disability [264]. CSWS has been reported even in β-Propeller protein-associated neurodegeneration, a neurodegeneration with brain iron accumulation due to a de novo WDR45 deletion at Xp11.23 [265]. Sager et al. reported one boy with a de novo loss-of-function mutation in the *TET3* gene located on chromosome 2p13 (Beck–Fahrner syndrome), revealed through whole-exome sequencing. The boy showed an early-onset neurodevelopmental delay, autistic behavior, ADHD, and learning disabilities. At 5 years of age he began to have generalized tonic–clonic seizures and clinical regression. An EEG showed ESES. He showed a clinical improvement while taking valproic acid and immunotherapy, regardless of EEG, which then improved with clobazam [266]. The CSWS EEG pattern has also been found in ferric chelate reductase 1-like (FRRS1L) encephalopathy. Recognizing ESES in these patients could be very important for proper management, although there are insufficient data regarding the real efficacy of the therapy [267]. The case with FRRS1L encephalopathy reported by Hadi responded well to the therapy with sulthiame for seizures, but not for CSWS [268]. Ünalp et al. described a child with Ehlers–Danlos syndrome and an epileptic encephalopathy as “ESES” related to an *STXBP1* (9q34.11) gene mutation, whose drug-resistant seizures stopped with KD [269]. We reiterate here the importance of paying particular attention to any cognitive and behavioral worsening that may be related to the CSWS EEG pattern in individuals with genetic pathologies, which are most often characterized by intellectual and behavioral disorders already present before the onset of CSWS.

Advances in genetics also help us find commonalities between clinically similar but distinct nosographic entities. The concept of the epilepsy–aphasia spectrum includes some epileptic syndromes (SeLECTS, atypical benign partial epilepsy (ABPE), LKS, and epileptic encephalopathy with CSWS) that have in common the association between epilepsy, language disorders, and centro-temporal spikes shown through EEG [241]. The theory of a spectrum that unites these conditions, suggested by the shared morphology of EEG paroxysmal abnormalities and by the possible evolution from one into another syndrome (e.g., SeLECTS into CSWS), is present in many works in the literature, albeit with some differentiations among authors [270,271,272,273,274,275,276,277]. The results obtained through genetic techniques such as copy number variation, exome sequencing, and linkage suggest a genetic etiology for these conditions [272,278,279]. In particular, mutations of the *GRIN2A* gene have been found in patients with focal epilepsy and speech disorders, including LKS [241]. There are data in the literature suggesting that patients with *GRIN2A* mutations are predisposed to a good response to immunotherapy, in particular using IV immunoglobulins [280]. According to Samanta, the discovery of *GRIN2A* as the main monogenic etiology of the epilepsy–aphasia spectrum led to a hypothesis on the possibility of precision therapies. Pathogenic variants of *GRIN2A* induce gains or losses of NMDAR function; possible therapies for the former are uncompetitive NMDAR antagonists (see, in particular, memantine), while a possible therapy for the latter is the NMDAR co-agonist serine. Precision therapies for *GRIN2A*-related disorders could benefit these patients [281]. 

Table 8 summarizes the etiopathogenetic factors of CSWS.

## 4. Discussion

CSWS is one of the most fascinating and complex topics in childhood epileptology of recent decades due to the various implications that characterize them: the role of epileptic seizures and epileptiform EEG paroxysmal abnormalities in determining a neuropsychological/behavioral disorder, the role of sleep in promoting learning, and the search for effective therapies without significant harmful effects on neurocognitive development and behavior.

A great heterogeneity of neuropsychiatric conditions associated with epilepsy with CSWS has been reported in the literature, including learning disorders, intellectual disability, ADHD, language disorders, and autism spectrum disorder. Note that CSWS have also been found in subjects with epilepsy, at least apparently in the absence of cognitive and behavioral deterioration, during a long-term follow-up (mean duration: 14 years) [282], but even in these cases, we cannot completely rule out that CSWS had a negative impact on neurocognitive development, as we do not know what results these individuals would obtain on neuropsychological tests in the absence of CSWS. Therefore, it cannot be stated that there is a characteristic neuropsychiatric picture associated with CSWS, except perhaps for the originally described classic ESES (with SWI ≥ 85%) (characterized by a global deterioration in cognitive functions) and LKS (characterized by a mixed-type acquired aphasia). This is also due to the fact that the pathogenetic role played by the EEG pattern of CSWS is often not at all clear in the patient’s history. For some cases who are initially completely or almost completely intact on the neurodevelopmental side and then show cognitive regression, this role appears to be preponderant, while in other cases, CSWS seem to be only a sort of EEG epiphenomenon that overlaps with an already heavily compromised neuropsychiatric picture. Particularly in patients who have an impairment in intellectual functioning that precedes the onset of CSWS, it may not be easy to identify the signs/symptoms that are indicative of cognitive deterioration related to the ESES picture. In these cases, the administration of standardized neuropsychological and behavioral tests is fundamental in order to have an objective evaluation of the individual’s situation. In fact, the clinical impression that can be obtained during an outpatient visit and the anamnestic data reported by caregivers are not sufficient and are sometimes unintentionally even misleading. 

All of this obviously has important repercussions with regard to the treatment of CSWS. While, on the one hand, treating CSWS in a child who presents neurocognitive regression or stagnation that is apparently related to CSWS is inevitable and not postponable, a very different situation is that of a child for whom the real pathogenetic value of CSWS is unclear. And in this regard, it should never be forgotten that even the drugs that can be used to treat CSWS are not without side effects, particularly (but not only) on the neuropsychiatric side. It may seem trivial, but it is worth underlining that our goal should not be to “clean” the EEG tracing during sleep at all costs, but to try to understand whether the CSWS pattern has a negative impact on the neurodevelopment of the child we have in front of us and, if so, try to treat it. The choice of whether or not to treat CSWS must be made considering the individual case as a whole, also taking into account the results emerging from the standardized neuropsychological and neurobehavioral tests that were administered. When faced with a patient with CSWS, it appears fundamental to carry out a series of examinations, particularly genetic tests and brain imaging, which, together with the previous medical history, can better outline the prognosis of the condition and can sometimes give useful suggestions regarding the decisions to be made on the therapeutic side. If, for example, we are dealing with a patient who has already suffered from epilepsy, we must first rule out a possible iatrogenic cause (in particular, the previous introduction of a drug that potentially favors the onset of CSWS). In this case, it may be sufficient to change medication. In other cases, which are clearly the most numerous, it is necessary to undertake a pharmacological treatment whose aim is the resolution of the sleep EEG pattern of CSWS and the improvement of cognitive skills in the absence of significant side effects. First, treatment with a benzodiazepine may be attempted, the effects of which on sleep EEGs can be assessed quite quickly. In cases of failure, or in cases of a relapse of CSWS, the best option seems to be a course of steroid therapy, which requires particularly careful medical monitoring. Furthermore, it should not be forgotten that therapeutic success is not constant, as CSWS represent an electro-clinical picture that often turns out to be drug resistant.

CSWS is an age-related condition that tends to disappear after adolescence, but especially when it has had a long duration, it can cause lifelong neuropsychiatric impairments. This is why we reiterate the importance of promptly performing, and possibly repeating over time, EEG tracing, particularly in sleep, in children with neurobehavioral problems, and in particular in those with unexplained neurodevelopmental regression or stagnation, even in the absence of overt epileptic seizures [148,283,284]. It is still to be ascertained whether the SWI is the best electrophysiological parameter to monitor the situation. Other parameters have also been studied and appear to be promising, in particular, HFOs, but they have so far had little use in daily clinical practice.

From a research perspective, we find interesting the point of view of Shao and Stafstrom, who reviewed CSWS and LKS animal models, underlining that they can reveal pathogenetic mechanisms that are potentially treatable with targeted therapies [285].

## 5. Conclusions

In conclusion, although several decades have passed since the original descriptions of the electroclinical condition of CSWS, there are still many areas that are little known and deserve to be further studied. We do not yet have a complete knowledge of the natural evolution of these conditions. There is not yet a shared approach among researchers regarding the nosographic classification of LKS, which, according to some authors, should be considered separately from ESES, while according to others, it represents a variant of it. It has not been fully ascertained whether the SWI is the most effective electrophysiological parameter for monitoring the evolution of the disorder. There is not yet a neuropsychological evaluation protocol that is shared internationally among the centers dealing with these problems. There is still no consensus as to whether to treat, and if so, when exactly to start drug treatment. We do not yet know a drug therapy that is effective for all cases of CSWS and free from significant side effects. We still know little about the non-pharmacological (medical, neurosurgical, and rehabilitative) therapies of these conditions. Finally, we believe that what Halasz underlined in 2017 is still valid today, at least in part; namely, we lack detailed knowledge of the nature (i.e., generalized or focal/regional?) of the EEG paroxysmal abnormalities in CSWS, and most EEG paroxysmal abnormalities show a morphology, spatial localization, and functional properties that are different from the generalized spike–wave pattern of typical ESES [286]. This is one of the main reasons for the great heterogeneity and complexity of these conditions in terms of diagnosis, prognosis, and treatment.

## Figures and Tables

**Table 1:** Clinical and EEG features of typical and atypical ESES.

	Clinics	EEG
Typical ESES	Cognitive deterioration. Behavioral disorders, including attention deficit, hyperactivity, aggressiveness, difficulty in social interaction, and (more rarely) psychosis; autistic behavior is also possible. Epileptic seizures, focal or apparently generalized, with a heterogeneous semeiology. Motor signs, including dyspraxia, ataxia, and dystonia.	Electrical-status epilepticus lasting for ≥85% of NREM (slow) sleep, documented by more than 2 EEG recordings during a period of ≥1 month.
Atypical ESES	Great heterogeneity in the reported clinical features depending on the frequency and localization of the EEG paroxysmal abnormalities: a cognitive deterioration has been reported, but less frequently than typical ESES. Behavioral disorders, in particular ADHD-like symptoms. Epileptic seizures, focal or apparently generalized, with a heterogeneous semeiology.	Paroxysmal abnormalities lasting for <85% of NREM sleep (usually > 50% < 85%), with a localization more heterogeneous than typical forms: focal, multifocal, unilateral, asymmetric or symmetric bilateral, and diffuse.

EEG: electroencephalogram. ESES: electrical-status epilepticus during slow-wave sleep. ADHD: attention-deficit/hyperactivity disorder.

**Table 2:** Predictive factors of the evolution into CSWS.

Clinical	Early onset of seizures; multiple types of seizures; appearance of new seizures with an increased frequency; seizure semiology including dysarthria or somatosensory auras.
EEG	Fronto-centro-temporal focus, with increasing frequency, both in wakefulness and in sleep; pattern EEG of spike–waves.

CSWS: continuous spike–waves during slow sleep. EEG: electroencephalogram.

**Table 3:** Electrophysiology and functional neuroimaging in CSWS.

Semiautomatic quantification of paroxysmal abnormalities in CSWS is a reliable alternative to the classic quantification based on visual scoring.
Global synchronization increase from wakefulness to sleep is strongly correlated with spikes.
Associating time-sensitive magnetic source imaging and PET, spike–wave onset is associated with focal hypermetabolism.
Magnetoencephalography in non-lesional CSWS children showed dipole clusters located on heterogeneous cortical areas: right Rolandic area, right supramarginal gyrus, left Rolandic area, left supramarginal gyrus, bilateral Rolandic area, and multiple anatomical areas.
PET showed hypermetabolism in perisylvian, superior temporal, inferior parietal, and central cortex areas that were related to paroxysmal abnormalities. The diffuse hypometabolism found in regions belonging to the DMN (see prefrontal and posterior cingulate cortices, parahippocampal gyrus and precuneus) could be due to remote inhibition following epileptic activity.
EEG-fMRI showed characteristic findings of epileptic encephalopathy: positive changes in BOLD signals in the perisylvian regions, prefrontal cortex, anterior cingulate, and thalamus, while negative changes in BOLD signals were found in the DMN regions. The activation pattern represents a diffusion of epileptic activity.
HFOs are hypothesized to be related to alterations in higher brain functions. They seem to be related to the functional disruption of brain networks in CSWS.

CSWS: continuous spike–waves during slow sleep. PET: positron emission tomography. DMN: default mode network. EEG-fMRI: electroencephalogram-functional magnetic resonance imaging. BOLD: blood-oxygen-level-dependent. HFOs: high-frequency oscillations.

**Table 4:** Non-surgical treatments for CSWS.

Pharmacological	Antiepileptic drugs: sulthiame (++), levetiracetam (+), acetazolamide (+), benzodiazepines (++), topiramate (++), perampanel (+), lacosamide (+). Steroids (corticosteroids and ACTH) (+++). Immunoglobulins (+). Amantadine (+).
Non-pharmacological	Ketogenic diet (+).
Comments on the literature concerning these topics: almost only retrospective studies were performed, with small or very small samples of patients; there is a lack of information about the long-term effects of the drugs. Often cognitive and behavioral findings reported do not derive from standardized objective neuropsychological assessments.	

CSWS: continuous spike–waves during slow sleep. (+): limited data (preliminary data resulting from studies with a limited number of patients). (++): consistent data (mostly coming from retrospective studies with a large number of patients). (+++): very consistent data (coming from retrospective or prospective studies with a large number of patients, on which there is broad convergence based on the literature).

**Table 5:** Main prognostic factors of CSWS.

Unfavorable	Long duration of CSWS. Presence of a cerebral lesion. High frequency of EEG paroxysmal abnormalities. Early-onset CSWS (before 6 years).
Favorable	Clinical phenotype of an atypical SeLECTS at onset. Normal development before CSWS. Later-onset CSWS (from 8 years onwards). Normal EEG background organization.

CSWS: continuous spike–waves during slow sleep. EEG: electroencephalogram. SeLECTS: benign epilepsy with centro-temporal spikes.

**Table 6:** Clinical and EEG features of LKS.

Clinical	Acquired mixed aphasia, with onset at 3–5 years of age, with poor decoding of verbal and/or non-verbal sounds. Seizures are present in around two-thirds of cases and are semiologically heterogeneous: focal motor, tonic–clonic seizures, and atypical absences. Behavioral symptoms are frequently associated: ADHD symptoms; irritability; aggressive behavior; in some cases, autistic-like symptoms; anxiety; and depression. Learning disorders are the rule. Outcome is very heterogeneous. Language recovery completes in only a minority of cases.
EEG	Spikes and spike–waves prevailing in the posterior temporal regions, bilaterally, much more diffuse and frequent during non-REM sleep, becoming continuous or subcontinuous. Background activity during wakefulness and sleep is normal.

EEG: electroencephalogram. LKS: Landau–Kleffner syndrome. ADHD: attention-deficit/hyperactivity disorder.

**Table 7:** Treatments for LKS.

Pharmacological	Antiepileptic drugs: valproate (+), benzodiazepines (+), levetiracetam (+), ethosuximide (+), acetazolamide (+). Corticosteroids (+++). Immunoglobulins in cases with GRIN2A mutations (++).
Non-pharmacological	Ketogenic diet (+). Multiple subpial transections in selected cases (+). Vagus nerve stimulation (+). Speech therapy (++).

LKS: Landau–Kleffner syndrome. (+): limited data (preliminary data resulting from studies with a limited number of patients). (++): consistent data (mostly coming from retrospective studies with a large number of patients). (+++): very consistent data (coming from retrospective or prospective studies with a large number of patients, on which there is broad convergence based on the literature).

**Table 8:** Etiopathogenetic factors of CSWS.

Brain structural alterations	Polymicrogyria (in particular, unilateral), migration disorders, perinatal hypoxic–ischemic encephalopathy, hydrocephalus, schizencephaly, porencephalic lesions, encephalitis, intracranial hemorrhage, and thalamic lesions.
Inflammation	Altered levels of interleukin-6.
Acquired factors	Fetal alcohol syndrome. Iatrogenic (in particular, old antiepileptic drugs).
Genetics	Dup15q (copy number gains of 15q11–q13), 22q11.2 microduplication, and many other copy number variants, affecting several other chromosomes, including 3q25 deletion, 3q29 duplication, 8p23.3 deletion, 10q21.3 deletion, 11p13 duplication, 16p13 deletion, and Xp22.12 deletion. Mutations of the *GRIN2A* gene (16p13.2). Mutations of a lot of other genes: *CNKSR2* (Xp22.12), *CDKL5* (Xp22.13), *CARS2* (13q34), *KCNQ2* (20q13.33), *KCNA2* (1p13.3), *SLC9A6* (Xq26.3), *HIVEP2* (6q24.2), *RARS2* (6q15), *DYNC1H1* (14q32.31), *FRRS1L* (9q31.3), *KCNA1* (12p13.32), *SLC9A6* (Xq26.3), *STXBP1* (9q34.11), *MECP2* (Xq28), *WAC* (10p12.1), *KANSL1* (17q21.31), *TET3* (2p13), *ZEB2* (2q22.3), and *WDR45* (Xp11.23).

CSWS: continuous spike–waves during slow sleep.

## Data Availability

No new data were created or analyzed in this study. Data sharing is not applicable to this article.
